# Coacervation as a Novel Method of Microencapsulation of Essential Oils—A Review

**DOI:** 10.3390/molecules27165142

**Published:** 2022-08-12

**Authors:** Alicja Napiórkowska, Marcin Kurek

**Affiliations:** Department of Technique and Food Development, Institute of Human Nutrition Sciences, Warsaw University of Life Sciences (WULS-SGGW), Nowoursynowska 159 c, 02-776 Warszawa, Poland

**Keywords:** complex coacervation, essential oils, nutraceuticals, proteins, mucilage

## Abstract

These days, consumers are increasingly “nutritionally aware”. The trend of “clean label” is gaining momentum. Synthetic additives and preservatives, as well as natural ones, bearing the E symbol are more often perceived negatively. For this reason, substances of natural origin are sought tfor replacing them. Essential oils can be such substances. However, the wider use of essential oils in the food industry is severely limited. This is because these substances are highly sensitive to light, oxygen, and temperature. This creates problems with their processing and storage. In addition, they have a strong smell and taste, which makes them unacceptable when added to the product. The solution to this situation seems to be microencapsulation through complex coacervation. To reduce the loss of essential oils and the undesirable chemical changes that may occur during their spray drying—the most commonly used method—complex coacervation seems to be an interesting alternative. This article collects information on the limitations of the use of essential oils in food and proposes a solution through complex coacervation with plant proteins and chia mucilage.

## 1. Introduction

Consumer interest in foods with high nutritional value, longer shelf life, and health benefits (functional food—FF) is growing year by year. This leads to the restriction of the use of preservatives and synthetic food additives in favor of bioactive substances of natural origin, e.g., from fruits, vegetables, and other plant sources. However, many of them are characterized by high instability—they are prone to oxidation, which is intensified by light, temperature, moisture, and changes in pH. The fashion for “healthy eating” and concern for the natural environment make food producers try to reduce or eliminate the addition of synthetic food preservatives and make them interested in technologies that allow the stabilization of bioactive substances to maintain their functional properties during processing and storage and to modify their physical properties to facilitate dosing [1,2,3].

## 2. Types of Functional Food

Food products known as functional, bioactive, enriched, modified, FOSHU (Foods for Specified Health Use), nutraceuticals, or food designed for the specific needs of the organism have appeared on the global market. The richness of the terminology used results from the variety of products classified as such food [4].

In the US, the Food and Drug Administration (FDA) defines functional food as food and its components that provide health benefits beyond their basic function. Similarly, in Canada, functional food is understood as food that, in addition to having basic nutritional functions, has a proven beneficial effect on health and/or reduces the risk of chronic diseases. In turn, in the European Union, since 1999, there has been a definition according to which functional food exerts a beneficial effect on one or more bodily functions in addition to its nutritional effect. The effect is to improve health and well-being and/or reduce the risk of diseases. It must resemble conventional food and it cannot be tablets, capsules, or dietary supplements. In addition, it is a requirement to prove the beneficial effect on the human body and the statement: nutritional and health. The concept of functional food is associated with the term enriched food, but these are not identical concepts. Fortified food means adding one or more nutrients to a food, whether or not they are naturally present in the food, e.g., yogurt with probiotics and margarine with phytosterol [4,5].

Nutraceuticals are an interesting concept. This term means ingredients isolated from food, dietary supplements, and herbal products that are used individually or combined to use their synergistic effects. They include biologically active substances with proven health-promoting properties, e.g., dietary fiber, proteins, lactic acid bacteria, antioxidant substances, etc. [4].

## 3. Trends among Consumers

Consumers nowadays are much more interested in information about the food products’ production method and ingredients production. Some production methods are perceived as less “natural”, and some food ingredients are perceived as “unhealthy” and “foreign” (i.e., artificial additives). This phenomenon, often referred to as the “clean label” trend, prompts the food industry to replace synthetic agents widely used in food with ingredients of natural origin, e.g., of plant origin [6].

Preservatives are used to prevent or inhibit unfavorable changes such as microbiological (growth of bacteria or fungi), chemical (oxidation, non-enzymatic browning), and biochemical (inactivation of certain enzymes, metabolites, and components necessary for the development of microorganisms), whereas food additives are substances that are added to food products to modify or improve their sensory qualities [2,7,8]. Unfortunately, more and more examples show that the consumption of artificial (chemical) additives and preservatives can lead to allergies, food poisoning, or the development of cancer. The most famous additives causing allergic reactions are sulfites (e.g., sulfur dioxide and sodium sulphite), which are traditionally used to preserve fruit and vegetable products. In addition, the addition of SO_2_ is a longstanding and common practice utilized to preserve the quality of wine (prevention against oxidation and browning) [9]. The significant reduction in vitamin B_1_ absorption caused by these compounds is responsible for the development of allergic reactions. Carmine and Cochineal Red are the other best-known food additives to cause allergies. These red pigments come from the bodies of female *Dactylopius coccus*, insects that grow on cochineal cacti (Central and South America, Southern Europe, and India). These dyes are widely used as colorants in processed foods and beverages. Most often it can be found in burgers, sausages, red alcohols, soft drinks, sweets, and fruit yogurts [10,11,12,13].

To meet the demands of consumers, food producers have taken great efforts to eliminate commonly used additives and preservatives from their products. In addition in recent years, more and more researchers have been developing methods that enable the use of natural substances for this purpose. For example, the use of antioxidants such as butylated hydroxytoluene (BHT), butylated hydroxyanisole (BHA), and tert-butyl hydroquinone (TBHQ) is not as popular in recent decades due to raised concerns about their adverse effects on human health. Even though it is believed that those substances are effective against oxidation reactions, substances of natural origin are sought for replacing them [14,15]. This, along with the traditional popularity of consuming natural products, has encouraged not only food producers but also scientists to explore the applicability and effectiveness of natural compounds such as essential oils as alternatives to harmful chemical antioxidants in food products [15]. Compounds derived from natural sources have great potential to extend the shelf life of food due to their antimicrobial properties against foodborne pathogens. However, this is not their only advantage. These types of substances can provide additional health benefits since they are very often bioactive compounds with antioxidant properties [9,10,12,13].

## 4. Essential Oils—Natural Preservatives and Functional Additives?

**Figure 1:**
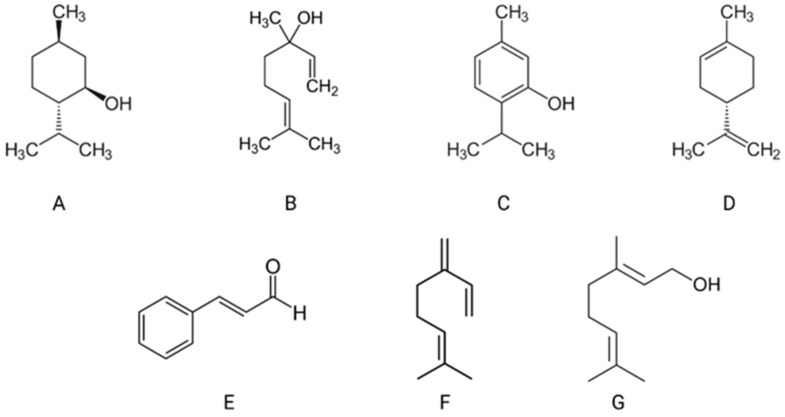
Examples of the main compounds of essential oils: menthol (**A**), linalool (**B**), thymol (**C**), limonen (**D**), geraniol (**E**), cinnamaldehyde (**F**), myrcene (**G**), own elaboration [21].

Essential oils (EO) are called essential in the sense that it contains the essence of the aroma of the plant it is derived from, whereas the term “oil” is used because it contains the oil-soluble chemicals in the plant, not only because it feels oily. EOs are also known as volatile oils, ethereal oils, or aetheroleum. Essential oils are secondary metabolites synthesized by oil-yielding plants. EOs are multi-component, hydrophobic mixtures containing up to several hundred volatile compounds (usually 100 to 200 chemicals per essential oil) in different concentrations. Essential oils can be characterized by two or three major components at relatively high concentrations (20–70%), which determines the biological properties of EOs (Figure 1). The main ingredients chemically are terpenes, aldehydes, ketones, phenols, alcohols, and others [16,17,18]. This complex chemistry gives them their therapeutic properties and explains why different essential oils may have overlapping effects [1,2,19,20]. Some examples of major components can be cited—carvacrol and thymol represent, respectively, 30% and 27% of the composition of EO from oregano (*Origanum compactum*). EOs from coriander (*Coriandrum sativum*) have in their constitution 65% linalool. Menthol (59%) and menthone (19%) are found in EOs from peppermint (*Mentha piperita*) [21,22].

Essential oils are characterized by having many pharmacological properties, including anti-inflammatory, antispasmodic, sedative, analgesic, and digestive-supporting properties. Due to their very rich and diverse chemical composition, one essential oil can have several positive effects. For example, rosemary essential oil has the effect of improving digestion, enhancing appetite, and being anti-flatulence. In addition, they have well-documented antimicrobial activity against bacteria, yeasts, and molds [1,2,3]. Again, thanks to its complex composition, one essential oil can effectively inhibit the growth of both bacteria and fungi. The same rosemary EO inhibits the growth of Gram-positive (*Enterococcus* spp.) and Gram-negative (*Salmonella* spp.) bacteria, yeasts (*Candida* spp.), and molds (*Penicillium* spp.) [23,24,25]. For this reason, EOs can be an alternative to the commonly used food-preservation agents.

This is indicated by studies conducted, inter alia, by Coimbra et al. [26]. The team tested the applicability of thyme essential oil (*Thymus zygis*) to *Listeria monocytogenes* in four food matrices (chicken juice, lettuce leaf model, ultra-high-temperature (UHT)-treated skim, and whole milk). EO inhibited the growth of *L. monocytogenes* 13305 in a model medium with chicken juice and lettuce. A significant reduction in the number was observed for the two highest concentrations of EO tested from 4 to 14 days for chicken juice and from 2 to 14 days for the model medium with lettuce. Research on the possibility of using essential oils for preserving food products was also conducted by Shah et al. [27]. Thymol concentrations used in apple cider resulted in complete bacterial inhibition or were bacteriostatic at 35 °C for *E. coli* and 32 °C for *L. monocytogenes*. Attempts have been made to use essential oils also for preserving such products as romaine lettuce, iceberg lettuce, mature bunched spinach, and baby spinach [28], snacks based on meat and seafood [29,30,31], juices [32], milk, yogurts, and other milk products [33,34,35] or chocolates [36], but also fruits or vegetables coated with edible coatings with the addition of EO [37,38].

Nanodispersion of eugenol (the basic ingredient of clove oil) in whey protein isolate and maltodextrin did not change its antimicrobial properties against *E. coli* O157: H7 and *Listeria monocytogenes*. However, nanodispersion allowed eugenol to be evenly distributed in the milk at concentrations above the solubility limit of the antimicrobial agent, which improved the antimicrobial efficacy in milk. Thus, nano-delivery systems hope to reduce the amount of antimicrobials without altering the turbidity of food products [33].

The stability of meat products during storage is a primary factor that is compromised by lipid oxidation and microbial growth. Hemmatkhak et al. [39] researched the use of active papers soaked in a nanoemulsion or Pickering emulsion containing cumin seed essential oil (CSEO). The effect of active papers on the quality and shelf-life of beef hamburgers stored at 4 °C for 7 days and at −18 °C for 60 days was investigated. Research results indicate good antioxidant and antimicrobial activity of cellulose papers impregnated in CSEO capsules. Packing beef burgers in contact with the produced active papers had a significant effect on extending the shelf life of hamburger samples by significantly reducing TBARS, the total number of mesophilic bacteria and psychrophilic. Furthermore, the sensory characteristics of the hamburgers did not changed.

It also seems important that many essential oils are on the Generally Recognized As Safe (GRAS) list published by the US Food and Drug Agency (FDA). Among the EOs that are approved for use in food are clove, rosemary, oregano, basil, mint, lavender, sage, cinnamon, and laurel [15,40,41]. Considering the above, essential oils can be not only a natural replacement for artificial preservatives but also a functional additive.

## 5. Microencapsulation as a Solution for EOs Application Limitations

**Figure 2:**
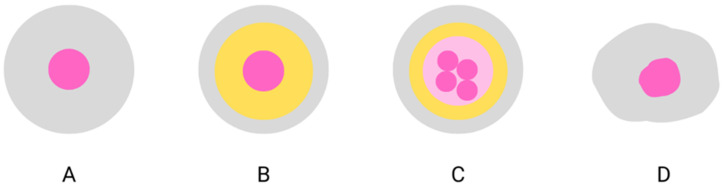
Schematic illustration of various morphologies formed by microencapsulation: monolayer and mononuclear microcapsule (**A**), multilayer and mononuclear microcapsule (**B**), multilayer and multinuclear microcapsule (**C**), and microparticle (**D**), own elaboration [46].

However, there are limitations to the use of pure essential oils in food products. First of all, their characteristic strong aroma and taste can cause undesirable organoleptic changes. In addition, EOs are very sensitive to the influence of the external environment —light, oxygen, and temperature. Other limitations are their lipophilic nature and hence low water solubility, low bio-accessibility, and bio-availability [18,42]. These problems can be solved by microencapsulation—an effective method of preserving the quality of sensitive substances. Microencapsulation is defined as a method of coating or encapsulating a given material or mixture of materials within the shell of a specific material or system. The substance that is encapsulated is called “active”, “encapsulate”, “payload”, or “core” and constitutes 30–99% of the total weight of the capsule. The core material can be a single substance or a mixture of solid, liquid, and gaseous forms. The enclosing polymer is called “shell”, “wall”, “matrix”, or “coating” [42,43,44]. The wall material is usually insoluble and non-reactive with the core material. The wall can be made of gums, proteins, lipids, and synthetic polymers. The wall material is generally applied as a liquid (solution, suspension, or molten material) to permit enrobing of the core material. Because the task of the shell is to protect the encapsulated substance, it should have excellent film-forming and barrier properties against oxygen, water, pressure, heat, and/or light [44,45]. A single microcapsule may have a round or irregular shape, depending on the method of producing the microcapsule, the type of active, and wall materials (Figure 2). The average size of the microcapsules is 100–500 µm [46]. Therefore, this process can provide many benefits to the use of EOs in food recipes, including protecting them from harsh conditions (light, shear, oxygen, moisture, heat, and others), improving their solubility and bioavailability, increasing their controlled release, and preventing their interaction with other food ingredients. This also allows for the reduction of volatilization of volatile substances, slowing mass transfer or modifying the physical properties of the core material. It reduces the evaporative loss of liquids and the reactivity of the core material, and extends the duration of its activity [43]. Encapsulation facilitates application by transforming the liquid into a solid phase, ensuring precise dosing, improving stability, and masking the encapsulated substance’s taste and/or smell. In addition, microencapsulation of essential oils can be a viable and effective approach to liquid food matrices with high water content by increasing their dispersibility. Facilitating the distribution of EOs in food areas where microorganisms thrive (water phase) and minimizing their particle diameter may also contribute to improving their antimicrobial properties. The smaller size of the molecules favors the migration and attachment to the bacterial cell walls [18,47]. Commonly used microencapsulation techniques are emulsification, spray-drying, coaxial electro-spray system, freeze-drying, coacervation, in situ polymerization, extrusion, fluidized-bed-coating, and supercritical fluid technology [42,44].

## 6. Spray Drying—The Most Commonly Used Method for Encapsulation of Essential Oils

Microencapsulation by spray drying is the oldest (has been used since the 1930s) and most common process used for microencapsulation in the food industry to preserve the physicochemical properties of volatile compounds such as essential oils. This method is most often used in the food industry due to its low production costs, large-scale production in a continuous mode, variety of encapsulating matrices, and adequate retention and stability of volatile compounds [22,42]. It consists of atomizing the emulsion in a drying medium at a relatively high temperature, which allows for quick evaporation of water and almost instantaneous encapsulation of the core material [42,44]. Microencapsulation with the use of spray drying is characterized by high retention of volatile substances during processing and their protection during storage. During this process, multinuclear capsules are formed in which the essential oil is distributed both inside and on the surface of the microcapsule, and thus volatile substances may be lost. This loss might occur during the process at three stages: during atomization, after the drop formation on the surface when a stable membrane has not been formed, and where the water inside the drop exceeds the boiling point and bubbles formed within the drop burst, cracking the surface and releasing volatiles [48]. Essential oils are substances highly sensitive to temperature. Ambient temperature crucially influences EOs stability; because of this, EOs may be degraded during this process. In general, chemical reactions are accelerated by increasing temperature (according to the Arrhenius equation). On this basis, van’t Hoff’s law states that a 10 °C increase in temperature approximately doubles the rate of chemical reactions. The degradation of essential oils by heat is a chemical phenomenon and can occur by various pathways, which can be broadly classified as oxidative degradation, cleavage of the C-C bond, elimination, hydrolysis, and thermal rearrangement. Under the influence of elevated temperature and due to their structural relationship within the same chemical groups, components of essential oils can easily transform into each other mutually, which may cause changes in their taste, smell, and antimicrobial activity [21,48,49]. For this reason, it seems legitimate to look for alternative methods for the spray-drying of essential oils. To reduce the loss of essential oils and the undesirable chemical changes that may occur during their spray drying, the use of complex coacervation seems to be an interesting alternative.

## 7. Simple and Complex Coacervation—What Is the Difference?

**Figure 3:**
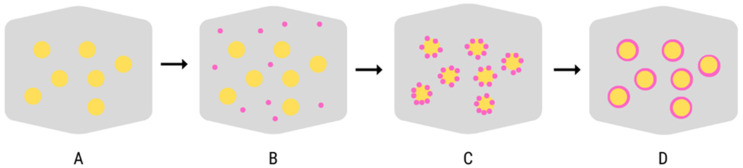
The mechanism of microcapsules formation in the coacervation method, liquid phase (**A**), suspending the core material in the liquid phase (**B**), deposition of liquid polymer around the core (**C**), gelling, and solidification of the microcapsule wall (**D**), own elaboration [43].

Coacervation is one of the oldest and most widely used encapsulation techniques. It is a relatively simple method that can be compared to a modified emulsification technique. Coacervation name comes from the Latin word *acervus*, which means aggregation, and the prefix *co* indicates the fusion of colloid particles [43]. The mechanism of this process consists of the separation of the hydrocolloid from the primary solution followed by agglomeration into a separate, liquid phase which is called “coacervate”. The coacervates are called the “continuous phase”, whereas the second phase is called the “equilibrium solution” [43,50]. The coacervation process can be divided into four stages: suspending the core material particles in the liquid phase, production of a three-phase system, i.e., secretion of the second liquid phase (coacervate), deposition of liquid polymer around the core, gelling, and solidification of the microcapsule wall (Figure 3).

Coacervation has been classified into simple (SC) and complex coacervation (CC). Simple coacervation refers to the cases where only one polymer is involved and salted out by the action of electrolytes (sodium sulfate) or desolvated by the addition of a water-miscible nonsolvent (ethanol) or by increasing/decreasing the temperature [50,51]. Complex coacervation is a phase separation process caused by the interaction of two or more oppositely charged colloids (biopolymers), usually proteins and polysaccharides. The term complex coacervation was first introduced by Bungenberg de Jong and Kruyt in the 1940s to distinguish it from simple single polymer coacervation [43]. In this technique, the liquid phase separates from the polymer-rich (coacervate) phase.

The main driving force for complex coacervation is the reduction in free electrostatic energy of the reaction system resulting from the interaction between oppositely charged ions [52]. This process is also influenced by parameters such as pH (coacervates formation occurs over a narrow pH range below the isoelectric point), ionic strength, protein-polysaccharide ratio, total biopolymer concentration, type of core material, and the core:wall ratio [42,48,53,54]. The speed of agitation plays an important role in controlling the size of the coacervates formed. In addition, the difference between the polymer loads must be large enough to cause interaction, but not large enough to cause precipitation. Highly charged molecules are reported to have an extended molecular conformation resulting in unfavorable coacervation [52]. Microcapsules prepared in this way are insoluble in water and heat-resistant but the main advantages of complex coacervation compared to other microencapsulation methods are the overall higher encapsulation efficiency and the possibility of using controlled release. The process results in a circular microcapsule in which the core is surrounded by a wall material that protects the active compound [48,53,54].

## 8. Wall Materials Used in Complex Coacervation

### 8.1. Gelatin and Arabic Gum—Standard in Complex Coacervation

The most common system used in complex coacervation is gelatin (G)–Arabic gum (AG) [54,55,56,57] in Table 1. It is recommended due to its abundance, biocompatibility, and biodegradability [36,38]. Two types of gelatin can be distinguished—A and B. Gelatin A is formed by partial hydrolysis of collagen in an acidic environment, whereas gelatin B is in an alkaline environment. The isoelectric point (pI) of gelatin A produced ranges from pH 6–9, whereas gelatin B possesses a pI of 4.8–5 [58,59]. During the complex coacervation, the electrostatic attraction between gelatin and the anionic polysaccharide (i.e., Arabic gum) occurs at a pH below 9 for gelatin A and a pH below 5 for gelatin B [59]. In the case of G and AG, the mechanism of complex coacervation can be explained by the electrostatic attraction between the positive protein charges (NH^3+^) and negative charges derived from AG (COO^−^) [59,60]. Gelatin and Arabic gum when exposed to electrostatic interactions form a coacervate layer that hardens in the process of gelatin cross-linking. In a chemically induced cross-linking process, the insoluble network is formed by the reaction of the aldehyde residues of the cross-linking agent and the amino groups of the protein. This newly formed network strengthens the wall of the capsule, thus facilitating the drying process and increasing the storage stability of the capsules [38]. However, this process is chemically induced by formaldehyde, glutaraldehyde, glyoxal, or epichlorohydrin, which are considered toxic to the human body, and are most often used to cause this process. This is one of the major limitations of the production of microcapsules for the food industry using the (G)–(AG) system [50,52,61]. This is not the only drawback of this combination. Preparation of a gelatin solution generally requires a relatively high temperature (50–60 °C) to completely dissolve the gelatin [52,56]. For that reason, the quality of sensitive compounds such as essential oil could deteriorate at this temperature. Another disadvantage of gelatin is its animal origin and, in the case of beef gelatin, its possible association with bovine spongiform encephalopathy (“mad cow disease”). Therefore, the most popular gelatin is that of porcine origin—not acceptable by a certain group of consumers based on their religious and dietary preferences [52]. Moreover, due to the increasing popularity of vegetarian and vegan diets, the current aim in the food industry is to minimize the use of ingredients of animal origin.

Arabic gum, also known as gum acacia, is a complex anionic polysaccharide with fractions of 90–99% arabinogalactan and 1% glycoprotein. This amazing composition gives it effective surface properties. Furthermore, it has a molecular structure with a galactan main chain carrying the highly branched galactose/arabinose side chains which contributes to a much higher negative charge density in comparison to a linear polysaccharide of the same composition. Moreover, Arabic gum has good cold solubility due to the presence of residual charged groups and peptide fragments [43,62] It also has low solution viscosity and the ability to form a protective film around emulsion droplets. All of this above makes Arabic gum an effective emulsifier and good encapsulating agent [43,63].

As already mentioned, the combination of G and AG is the most commonly used combination of wall materials in complex coacervation. After pioneering systematic studies of the complex coacervation of gelatin and acacia in 1949, the first practical application of this system was the microencapsulation of dyes [43,64]. Since then, a huge amount of work on the microencapsulation of various active substances using the complex coacervation of this pair of polymers as one of the leading applications of protein-polysaccharide coacervation in encapsulation technology has been studied over the last few decades. Coacervation between G and AG is induced with an aqueous solution of both polymers at pH 6–7 and a temperature of 50–60 °C, above the gelatin gelling point. This system has already been used to encapsulate various types of flavors and colorants [65], oils [50,54,66,67], food ingredients [56], and medicines [68]. It has also been used for the complex coacervation of essential oils. A research team led by Lv et al. [62] confirmed that the pH of 4.80 and the mixing ratio 1:1 between G and AG were suitable for the preparation of spherical nanoparticles with trapped jasmine essential oil. Analysis of structural properties and volatile flavor compounds showed that such nanocapsules have good heat resistance capability against humid heat (80 °C). The results suggested that the G-GA system may have potential use as a delivery vehicle for functional ingredients food. The results of another study examining the G-GA system [69,70] showed that the obtained coacervate microcapsules can be used for the sustainable release of EOs during food storage and as promising organic preservatives to inhibit foodborne pathogens.

### 8.2. Milk Proteins and Polysaccharides

Milk proteins (MP) have been extensively used in the food industry because of their amphiphilic nature, which allows them to adsorb and spread around the oil/water matrix. Those proteins are also popular as food additives for their nutritional, functional, and active properties. Milk proteins can be divided into whey proteins (WPI) (alpha with pI ranges from 4.3–4.7 and beta lactalbumin, pI = 5.2) and caseins, with pI ranges from 4.9–5.6 [59,71,72]. Many authors [71,72,73,74] have conducted complex coacervation with the use of milk proteins and various types of polysaccharides. From the research carried out so far, it is possible to conclude that increasing the concentration of MP and biopolymer ratio leads to an increase in the pH at which the CC process occurs including. This, in turn, leads to an increase in the average size of the microcapsules formed. This phenomenon can be explained by the decreasing force of electrostatic repulsion between proteins and polysaccharides [59]. The limitation in the use of milk proteins in the coacervation process is the fact that during the preparation of emulsions (ultrasound, temperature, high pressure), their partial denaturation and conformational changes may occur. This negatively affects the process of coacervate formation. As already mentioned, the food industry is trying to reduce the use of animal products. In addition, milk proteins are strong allergens [75].

Arabic gum is used as a polysaccharide for complex coacervation, along with milk proteins, e.g., chitosan, carrageenan, or alginates, which are also often used.

Chitosan (CH) is a cationic polysaccharide produced by the deacetylation of chitin in the hydrolysis of acetylamino groups in a highly alkaline environment and at elevated temperatures. Its structure consists of D-glucosamine and N-acetyl-D-glucosamine, linked by β (1 → 4) O-glycosidic bonds. Its low toxicity and allergenicity, as well as hydrophobicity, biodegradability, tissue biocompatibility, and antimicrobial activity, allow it to be used in edible film formulations or microencapsulation of bioactive compounds [76]. A study conducted by Tavares et al. [76] aimed to encapsulate garlic aqueous extract by complex coacervation between WPI and CH. FTIR analysis confirmed that garlic compounds were intact and encapsulated. Scanning electron microscopy images showed all microparticles with a spherical shape and no evidence of cracking or fissures on the surface. Therefore, it can be concluded that the combination of WPI and CH is a good alternative for use as wall systems to protect the bioactive compounds.

Carrageenan (CG) is the general name for a group of high molecular weight sulfated anionic polysaccharides extracted from red seaweeds formed by alternate units of D-galactose and 3, 6-anhydro-galactose (3, 6-AG) joined by α-1, 3, and β-1, 4-glycosidic linkage [72,77]. There are three main types of carrageenan, called kappa (*κ*), iota (*ι*), and lambda (*λ*). They are differentiated based on the number and position of sulfate groups on the galactose/anhydrogalactose chain. *κ*-carrageenan contains one sulfate group, whereas *ι* and *λ* have two and three per disaccharide repeating unit, respectively [72,78]. Since CG is a sulfated polysaccharide, it has a negative net charge in a wide pH range, which allows it to interact with positively charged compounds such as WPI under its isoelectric point where the protein has a positive net charge [72]

Alginates are natural polysaccharides isolated from the cell walls of various species of brown algae. They consist of linear chains (1–4)-connected b-D-mannuronic acid residues and A-L-guluronic acid in various proportions [79]. Bastos et al. [80] conducted extensive research on the microcapsule structure of black pepper essential oil (BPEO) obtained in the process of complex coacervation between lactoferrin (LF) and sodium alginate (SA). The authors indicate that the LF-SA system resulted in high encapsulation efficiency (>80%), and the essential oil components were retained. The main identified component of BPEO was β-caryophyllene. After the encapsulation process, 97.5% of this compound was protected. In addition, the researchers presented the results of studies obtained with the use of an artificial gastrointestinal tract. The black pepper EO capsule demonstrated resistance under oral and gastric conditions and release in the intestine, contributing to absorption in the in vitro simulation.

An interesting study was also conducted by Rojas-Moreno et al. [81] to compare the microencapsulation of orange essential oil by complex coacervation with whey protein isolate (WPI) and different polysaccharides: carboxymethylcellulose (CMC), SA, and CH. The process was successfully performed with an encapsulation efficiency of 94% (WPI:CMC), 88% (WPI:SA), and 91% (WPI:CH). Another study conducted by Soliman et al. [82] showed that EO microcapsules (thyme, cinnamon, cloves) made with calcium alginate can retain 30–50% of the antifungal activity of EO after a storage period of 8 days, whereas all of these EOs have lost all their antifungal activity after two days of storage.

### 8.3. Plant Proteins and Polysaccharides

Due to the above-described trends among consumers and the limitations connected with the use of gelatin and milk proteins for the process of complex coacervation, plant-origin proteins were of growing interest. Another reason is they are environmentally friendly, low cost, available, and have interesting functional properties [45]. Among various plant proteins, soy protein (SP) is the most well-studied and most commonly employed in the microencapsulation technique. This is because SP has functional properties for encapsulation, such as emulsification, solubility, film-forming, and water binding capacity, in addition to presenting high nutritional value (contains at least 90% protein; it is thus virtually free from lipids and carbohydrates) [83]. Due to their amphipathic nature (hydrophilic and hydrophobic), these proteins exhibit a good ability to diffuse and/or adsorb, and stabilize the interface of, oil droplets during emulsification, thus acting as effective emulsifiers to form and stabilize oil-in-water emulsions [84]. These properties of SP make it widely used in the microencapsulation process. However, the hydrophilicity/hydrophobicity balance of a protein’s surface is also thought to impact protein solubility, and this is crucial for the process of comprehensive coacervation. Soy protein is characterized by low solubility, but it can be increased by adding a polymer. Complexation-enhanced protein solubility is well observed in the literature [43,48,50,73,85]. It has been identified that the biopolymer mixing ratio is the factor most responsible for the solubility of proteins when they are in complexes. Significantly improved protein solubility for mixtures of soy protein isolate and xanthan gum at mixing ratios of 1:1 to 1:4 was observed [85]. Many research teams have confirmed that the use of soy protein together with polymer results in good stability of the core material during storage [70,86] or high process yield and the encapsulation efficiency [70,83].

Yuan et al. [70] conducted a study in which they proved that the use of soy protein and chitosan as wall materials for the complex coacervation of algae oil is effective in reducing its oxidation during storage. In similar studies [87] of the same SPI-CH system, DSC thermograms revealed increased denaturation temperature of SPI from 78 to 85 °C and elevated network thermal stability from about 38 to 43 °C.

However, proteins such as peas (PP), rice (RP), lentils (LP), or wheat (WP) are becoming popular in recent years. They possess different molecular weights and isoelectric points depending on the extraction method and plant source. As a result of using them instead of those of animal origin, the coacervation process can take place at room temperature. Yellow Pea (*Pisum sativum* L.) isolates are attractive for food and nutraceutical applications among plant proteins. This is due to their health properties, and the fact that PP is not allergenic and is gluten-free. In addition, PP is characterized by wide availability and low price [88].

The interactions between PP and different polysaccharides were applied (AG, tragacanth gum—TCG, tara gum—TG) to the microencapsulation of α-tocopherol by spray drying of complex coacervates. The effect of the protein/polysaccharide ratio was demonstrated—an increase in the proportion of polysaccharides increased the size of the particles in the suspension. In the presence of proteolytic enzymes, the PP-TCG mixture retained a stronger gastroprotective effect compared to the PP-AG matrices. The results of this study demonstrated the ability of PP to bind to plant polysaccharides, especially gum tragacanth, to form an interesting microencapsulation coating that is resistant to gastric digestion [89].

The influence of pH on the course of the complex coacervation process and the wall:core ratio on the physicochemical properties of the produced microcapsules have already been investigated [90]. The results indicate that the pH of the coacervation formation had a great influence on the microstructure of the coacervates. As a result, the technical characteristics of microencapsulation such as powder yield (PY), encapsulation efficiency (EE), and oil distribution in the microcapsules, as well as the oxidative stability of the encapsulated oil, dictated different results. PP and sugar beet pectin (SBP) were used as wall materials, and hemp seed oil (HSO) was the core material. Microcapsules spray dried from PP-SBP coacervates at pH 3.5 showed lower EE than those at pH 2.5. However, holes and/or partially broken particles were observed in the spray-dried microcapsules prepared at pH 2.5 (SEM observation), which had the effect of deteriorating the protection against oxidation of the encapsulated oil. Thus, the choice of the wall:core ratio and the pH of the coacervation formation is extremely important and should be determined by taking into account the balance between technical performance and the oxidative stability of the core material [Tab. 1]. Further research into pea protein as wall material is advisable.

At present, there are no studies on the use of PP or any other plant protein for the complex coacervation of essential oils. Nevertheless, it is known that they are able to form microcapsules in the process of complex coacervation, increasing oxidative and thermal stability of core substances. They can be successfully used for the controlled release of the core material and as a delivery system for the active ingredient. Therefore, we believe that complex coacervation using plant proteins has a bright future ahead of it.

### 8.4. Mucilage Instead of Commonly Used Polysaccharides

It is not only animal proteins that can be a starting point for the discussion. Additionally, Arabic gum, widely used in CC, which, despite its natural origin, has recently been negatively perceived by consumers. This is because AG is assigned the symbol E. The consumer approach has led scientists to try to replace Arabic gum with polysaccharides derived from mucilage raw materials such as chia seeds (*Salvia hispanica*), cress seeds (*Cardamine*), flax seeds (*Linum usitatissimum*), marshmallow (*Althaea officinalis*), aloe vera, prickly pear (*Opuntia ficus-indica*), etc. [91,92,93,94]. The combination of gelatin and chia mucilage results in a high encapsulation efficiency (>90%) of the essential oil [93].

Chia (*Salvia hispanica* L.) was eaten centuries ago as a staple food by the Mayas and Aztecs of Central and North America. It fell into oblivion after the Spanish conquest and is now experiencing its renaissance. It can be found in many different products, mainly for breakfast-bread, rolls, muesli, yogurts, ready-to-eat porridge, or smoothies. Chia seeds have a unique nutritional profile, hence the increase in popularity. Chia is an excellent source of ω-3 and ω-6 fatty acids, proteins with high biological value, antioxidants, vitamins, and minerals. In addition, it has been reported that the consumption of seeds is able to prevent inflammation and the occurrence of civilization diseases [95,96,97,98]. What is most important, chia seeds are capable of absorbing large amounts of water through swelling. Upon hydration, a hydrogel network is formed—soluble fiber, known as chia seed mucilage (CM)—which is and can be used in emulsification and foaming processes. Chia seed polysaccharides consist of, inter alia, D-xylose, D-glucose, β-d-xylopyranosyl acid, α-d-glucopyranosyl, and 4-O-methyl-α-d-glucopyranosyluronic acid [96,99]. CM is a promising material for the food industry because like popular vegan thickeners such as alginate, polyvinyl alcohol, and carrageenan, CM is biodegradable and digestible [95,100]. Consumption of CM has a positive effect on health by facilitating the passage of the contents in the intestines, thanks to which the peristalsis is improved. Additionally, CM has prebiotic properties [100,101].

Features such as appearance, color, flavor, texture, taste, aftertaste, and overall acceptability of orange juice with the addition of microencapsulated linseed oil with chia mucilage as a wall material were tested [102]. The results of the research showed that the enrichment of orange juice with nanoparticles did not affect the sensorics. Chia mucilage microcapsules have proved to be a good material for concealing the taste and smell of the encapsulated core substances. Similar studies were carried out for pomegranate juice that was fortified with microencapsulated fish oil. A complex coacervation between gelatin and acacia was used for microencapsulation. The study showed a significant decrease in the acceptability of oil-enriched juice compared to pure juice [66,102] The results of this study also showed that the nanocapsulation of linseed oil with chia mucilage as structuring material protected the linseed oil from oxidation. When it comes to in vitro digestion, good bioaccessibility to linseed oil has been observed, suggesting that the nanoencapsulation gave the oil digestive stability and thus the potential for use in food [102].

A similar study was conducted with microencapsulated chia seed oil (CSO) [103]. The study compared oil release from emulsions, simple coacervates of chia protein (CSP) or chia gum (CSG), and complex coacervates between CSP-CSG. Complex coacervate shell provided higher resistance to the release and subsequent digestion of CSO compared to that by CPI only or CSG-only matrices, indicating that protein-polysaccharide complex coacervates can preferably be used as shell materials for delivering sensitive food ingredients to the intestinal digestion stage with minimal damage in the harsh gastric environment. Such a combination could also be successful with essential oils. However, there are no studies available at the moment. Hernandez Nava et al. [93] conducted a study showing that the complex coacervation between gelatin and chia mucilage resulted in high encapsulation efficiency (>90%). Further research into the use of chia mucilage as a wall material for encapsulating essential oils is advisable.

**Table 1:** Encapsulation efficiency depends on the wall materials used, and its own elaboration.

Wall Material		Wall Material Ratio	Core Material—Essential Oil	Wall:Core Ratio	pH	Method of Emulsification	Encapsulation Efficiency [%]	Reference
Gelatin	Arabic gum	1:1	Citronella	2:1	4.5	Magnetic stirrer + cross-linking	94–42	[101]
Gelatin	Arabic gum	1:1	Geraniol	2:1	4.2	Cross-linking + high-speed homogenization	71–77	[57]
					4.45		87–91	
Gelatin	High methyl pectin	3:1	Peppermint	1:2	4.23	Magnetic stirrer + cross-linking	75–82	[61]
Whey protein isolate	Arabic gum	1:1	Ginger	1:3	3.66	High-speed homogenization + ultrasonication	5	[104]
		2:1					30	
		3:1					43	
		4:1					38	
		5:1					30	
		6:1					28	
		7:1					25	
Pea protein isolate	Arabic gum	2:1	No core material		2.5	No treatment	88	[76]
					3.0		95	
					3.5		99	
					4.0		91	
					4.5		90	
		5:1			2.5		70	
					3.0		90	
					3.5		92	
					4.0		98	
					4.5		92	
		10:1			2.5		78	
					3.0		88	
					3.5		94	
					4.0		98	
					4.5		92	
Gelatin	Chia mucilage	1:2	Oregano	1:1	3.6	Ultrasonication	91–79	[93]
Whey protein isolate	Quince mucilage	7:3	No core material		4.0	Magnetic stirrer	80–67	[74]

## 9. Complex Coacervation of Essential Oils

The process of microencapsulating essential oils by complex coacervation occurs in stages. The first one is hydration and preparation of wall materials solution. The essential oils and an emulsifier (e.g., Tween 20, 60, 80) are then added to these solutions, and the emulsification process is carried out. This step is essential because the quality of the emulsion and the interaction between its droplets directly affect the stability of the microcapsules produced later. The final step is curing by spray drying or freeze-drying [59,93].

**Figure 4:**
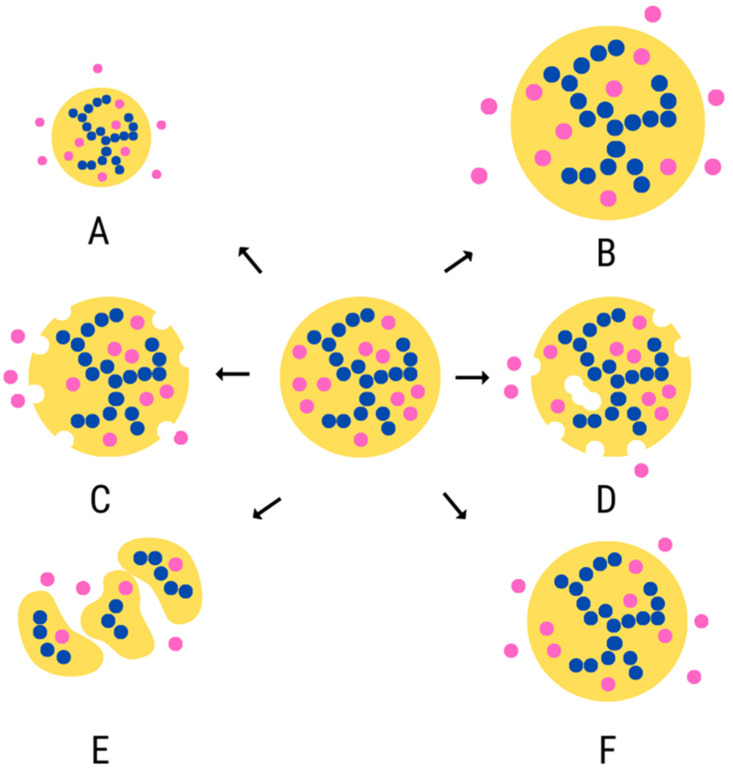
Various types of release mechanisms for EO-encapsulated component: shrinkage (**A**), swelling (**B**), surface erosion (**C**), bulk erosion (**D**), fragmentation (**E**), diffusion (**F**), own elaboration [106].

Microencapsulation of essential oils using complex coacervation allows for their controlled release. It is the process of delivering EO delayed after administration or incorporation into the food matrix for an extended period [59,105]. This process is influenced by environmental conditions (type of food matrix), type of EO, the composition of microcapsules (proteins, polysaccharides), and microcapsule architecture. Essential oils can be released from their microcapsules through a variety of mechanisms (Figure 4). The swelling mechanism involves increasing the pore size as the matrix swells, which promotes the release of the encapsulated Eos. The mechanism of erosion is the dissolution of the outer part of the support (surface erosion) or all of the support (bulk erosion), often due to enzymatic or chemical hydrolysis. The mechanism of fragmentation is the rupture or breakage of the support matrix, which often occurs due to mechanical forces. The resulting increase in surface area and shorter diffusion paths mean that the bioactive agents are released from the fragments faster than the original carrier. The diffusion mechanism involves the diffusion of the bioactive component through the carrier matrix into the surrounding environment. EO microcapsules should be designed to be used in a specific product because food is exposed to a variable temperature, ionic strength, pH and mechanical conditions and stress during processing and storage [76,104,106,107]. The preparation of microcapsules of essential oils produced by complex coacervation can be an effective method to preserve their physicochemical properties. At the same time, it can contribute to increasing the applicability of essential oils in food as natural additives with a preservative effect and increasing the nutritional value of the final product. EO microcapsules can be an effective way to reduce the use of synthetic food additives while enabling the creation of interesting products placed in the functional food segment.

## 10. Conclusions and Future Perspectives

The food industry is trying to move away from the use of artificial additives and preservatives in food. Currently, substances of plant origin, including essential oils, are gaining more and more popularity. Despite the strongly documented pro-health and antimicrobial properties, the use of essential oils on a larger scale is currently not possible due to their very strong taste and aroma, which negatively affects the acceptability of the products in which they are found. In addition, they are characterized by high instability (sensitivity to light, oxygen, and temperature) and a hydrophobic nature, which prevents their solubility in the water phase of food where microorganisms develop. Therefore, scientists are looking for solutions that will preserve their properties during storage and at the same time mask their strong taste and smell and reduce their hydrophobicity.

All these limitations may be solved by microencapsulating EOs using complex coacervation. It is an alternative method to the most commonly used spray drying. It allows not only to eliminate the elevated temperature during the encapsulation process, but also to better enclose the core material and its protection from the external environment. Additionally, the fact that microcapsules of essential oils obtained through complex coacervation would constitute a kind of nutraceutical deserves attention. The use of non-allergenic proteins of plant origin (peas, rice) and polysaccharides from chia seeds with properties improving intestinal motility would significantly increase the nutritional value and health-promoting effect of the product to which microcapsules prepared in this way would be added.

Future research should focus on the possibility of producing microcapsules of essential oils as described. There is also no information on the in situ use of essential oils as antimicrobial agents.

## Data Availability

Not applicable.
